# Estimation of Wind Speed Based on Schlieren Machine Vision System Inspired by Greenhouse Top Vent

**DOI:** 10.3390/s23156929

**Published:** 2023-08-03

**Authors:** Huang Li, Angui Li, Linhua Zhang, Yicun Hou, Changqing Yang, Lu Chen, Na Lu

**Affiliations:** 1 School of Building Equipment Science and Engineering, Xi’an University of Architecture and Technology, Xi’an 710055, Chinaluna@xauat.edu.cn (N.L.); 2 School of Thermal Energy Engineering, Shandong Jianzhu University, Jinan 250101, China

**Keywords:** schlieren system, SURF feature matching, fluid imaging kinematics, greenhouse, single vent ventilation

## Abstract

Greenhouse ventilation has always been an important concern for agricultural workers. This paper aims to introduce a low-cost wind speed estimating method based on SURF (Speeded Up Robust Feature) feature matching and the schlieren technique for airflow mixing with large temperature differences and density differences like conditions on the vent of the greenhouse. The fluid motion is directly described by the pixel displacement through the fluid kinematics analysis. Combining the algorithm with the corresponding image morphology analysis and SURF feature matching algorithm, the schlieren image with feature points is used to match the changes in air flow images in adjacent frames to estimate the velocity from pixel change. Through experiments, this method is suitable for the speed estimation of turbulent or disturbed fluid images. When the supply air speed remains constant, the method in this article obtains 760 sets of effective feature matching point groups from 150 frames of video, and approximately 500 sets of effective feature matching point groups are within 0.1 difference of the theoretical dimensionless speed. Under the supply conditions of high-frequency wind speed changes and compared with the digital signal of fan speed and data from wind speed sensors, the trend of wind speed changes is basically in line with the actual changes. The estimation error of wind speed is basically within 10%, except when the wind speed supply suddenly stops or the wind speed is 0 m/s. This method involves the ability to estimate the wind speed of air mixing with different densities, but further research is still needed in terms of statistical methods and experimental equipment.

## 1. Introduction

**Figure 1:**
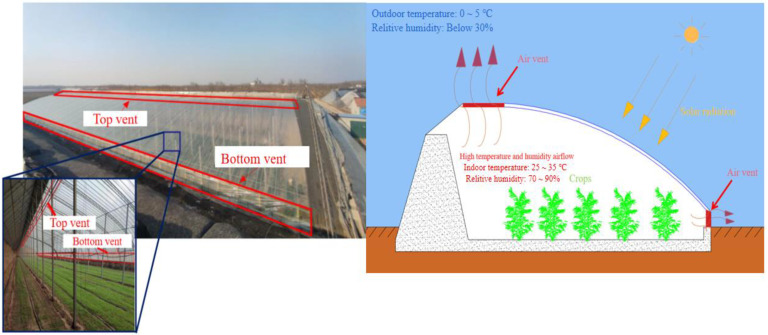
Single-slope greenhouse panorama.

Research on climate testing facilities and methods in greenhouses is significant for agricultural activities. Due to the high humidity in the greenhouse, which lead to plant disease, staff will open the ventilation openings of the greenhouse for ventilation and dehumidification [1]. In a single-slope greenhouse, the indoor temperature remains around 30 °C, although the external temperature is below 5 °C during the day. However, without opening the vents, its internal relative humidity reaches almost 100%, which poses a serious threat to crop health and growth. Greenhouse staff need to measure the wind speed at the vent to determine the ventilation volume. An appropriate ventilation amount ensures that the temperature inside the greenhouse drops slowly without cold leading to plant death, even if the air vent involves a certain moisture removal capacity [2]. During the actual investigation, to prevent the temperature from declining rapidly inside the greenhouse, the staff merely opened the top vent for natural ventilation. The vent part produces different natural ventilation effects according to the difference in external wind speed and wind direction. Airflow through the vent provides heat transfer and gas exchange inside and outside the greenhouse [3,4]. Therefore, a method for estimating the airflow velocity of high-temperature and humidity air flowing into low-temperature and humidity environments significantly helps workers adjust greenhouse ventilation strategies in a timely manner. Many scholars have tested and studied the microenvironment inside agricultural greenhouses based on multiple sensors [5,6,7]. However, there is a lack of research on the form of single-side ventilation in the horizontal direction. The structure of the single-slope greenhouse is shown in Figure 1.

**Figure 2:**
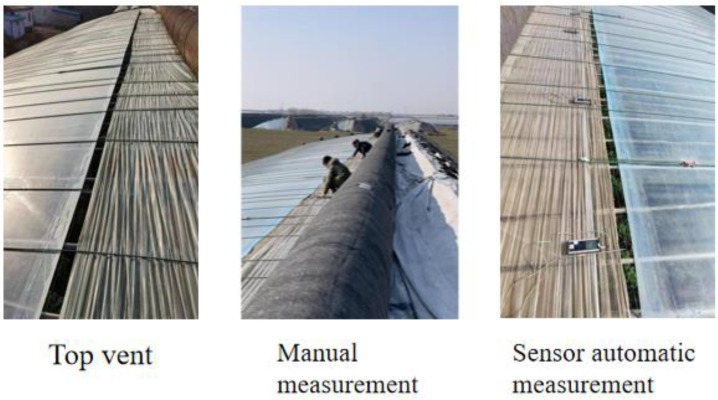
Greenhouse top vent and traditional measurement methods.

Traditional facilities for agricultural wind speed measurement mainly include hot-wire anemometers, cup anemometers, and ultrasonic anemometers. The hot-wire anemometer has small thermal inertia with a fast response [8]. However, the hot-wire type is unable to record the air-flow pattern and lacks the ability to against temperature change. A cup anemometer is a standard facility and is widely used for most greenhouse wind speed tests [9]. The structure of the cup anemometer includes four or two wind cups. Each cup is installed at one horizontal arm, which is on the vertical axis at an equal angle [10,11]. Therefore, the wind cup anemometer has complex rotating structures. For instance, the DEM6 three-cup anemometer has 21 components [12]. Additionally, the function of the cup anemometer is only used to measure the two-dimensional wind speed in the horizontal direction. It collects data from the sensor, which obtains the rotation rate of four hemispherical cups and calculates the wind speed. In the application environment discussed in this paper, it is necessary to monitor the wind particle displacement of the horizontal opening vent. Traditional measurement methods are shown in Figure 2. In this measurement scenario, it is not suitable to use this anemometer.

Meanwhile, through the functional relationship between rotation and wind speed, a cup anemometer is used as a low-pass mechanical integrator [13] with shielding turbulence and gust signals. Obviously, this monitoring method easily causes large errors. To overcome the disadvantages of cup-type anemometers, scientists have created ultrasonic anemometers [14]. Ultrasound anemometers are also widely used in agriculture with nonmoving parts on sensor detectors [15,16]. The mechanical motion mechanism is nonexistent within system parts, so ultrasonic transducers have the ability to receive a high-pass signal with a high sampling rate from airflow or a low-pass signal after the calculation of data [17,18]. However, the use of a precise structure and expensive sensors will inevitably result in higher expenditure and maintenance costs, and the initial cost generally exceeds EUR 2 k [13]. The high cost and harsh application occasion have become obstacles for greenhouse operators to purchase such equipment.

To alleviate the above problems, more machine vision methods are applied in the field of wind speed measurement. The dominant method is optical flow velocimetry (OFV). Researchers have found that the velocity of rigid body motion in continuous images is related to the light flow [19]. Based on the assumption that the light intensity of continuous images is constant, the intensity limit equation and the corresponding variogram equation are presented. Also, optical flow combined with PIV or devices for measuring fluid has been widely used and studied [20]. Linxin S and Tianshu L [21] correlated fluid imaging with OFV and proposed an optical flow equation based on fluid dynamics. Suter D [22] introduced the smoothing constraint form of second-order divergence-rotation space, which adjusts the relative gravity of divergence and rotation terms according to the motion characteristics, thus retaining the fluid characteristics well. Although many scholars have discussed the determination of fluid velocity by OFV completely, there are still some defects in the determination of thermal plumes and thermal turbulent jets. The light intensity invariance theory on which OFV depends is challenged in an open, non-adiabatic environment. The density gradient of hot plumes and thermal turbulent jets varies with temperature and pressure. This is contrary to the basic theory of light intensity invariance in optical flow. Therefore, a fluid velocity measurement method independent of the fluid density gradient is required. This paper mainly introduces a schlieren system with the SURF feature point matching method for wind velocity measurements for horizontal vents of single-slope greenhouses.The basic theory of this article is that the three-dimensional motion expression of fluid micro-clusters is mapped to the pixel changes of the fluid micro-cluster image on the imaging plane based on Helmholtz motion decomposition law. Meanwhile, the SURF feature matching algorithm is used to get the feature points of the morphologically processed image to match and quantify the displacement.

## 2. Principle and Experiment

### 2.1. Principle

In this paper, multiple moving images of each two adjacent frames are used from the schlieren system. The SURF feature extraction algorithm extracts feature points after image morphological analysis. This feature point is a fluid microcluster with the characteristics of an image convolution kernel. The image is matched to find the displacement vector of the fluid microcluster. The airflow velocity and direction are estimated by statistical calculation of the displacement value of characteristic points.

Within a very short period of time between adjacent frames, the morphology of the boundary of the fluid density gradient changes slightly. The SURF method will be able to match fluid image microclusters with the same boundary morphological characteristics from motion frame difference images. The SURF feature detection algorithm was proposed by Bay et al. [23]. in 2006 and 2007. The SURF feature matching method has reliable performance in feature point extraction and image calibration [24,25]. This method selects POI (points of interest) points by the Hessian matrix (Equation (1)). The Hessian matrix is calculated for each pixel to detect features. The matrix measures the local curvature of a function.

(1) $$H(x,y)=\left[\begin{array}{cc} \frac{\partial^{2}I}{\partial x^{2}} & \frac{\partial^{2}I}{\partial x\partial y}\\ \frac{\partial^{2}I}{\partial x\partial y} & \frac{\partial^{2}I}{\partial y^{2}} \end{array}\right]$$

**Figure 3:**
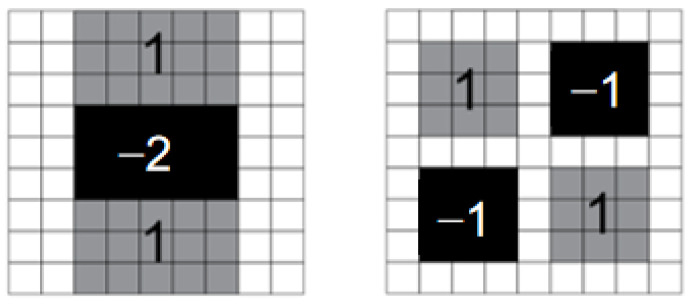
The approximate Gaussian kernel used in the literature.

According to the value of the Hessian matrix, the intensity of curvature can be obtained. The corners are defined as pixels with local high curvature. The surf feature detection algorithm makes use of the characteristics of the integral image so that the amount of calculation is independent of the image size. At the same time, the convolution pixel kernel uses an approximate Gaussian kernel which is shown in Figure 3.

The scale space function $D_{xx}$, $D_{yy}$ and $D_{xy}$ is obtained by using the Gaussian kernel and integral image. Then, the local extreme point can be obtained through the approximate expression of Δ(H) proposed by Bay et al., as shown in the following equation (Equation (2)):

(2) $$\Delta (H)=D_{xx}D_{yy}-{\left(0.9D_{xy}\right)}^{2}$$

**Figure 4:**
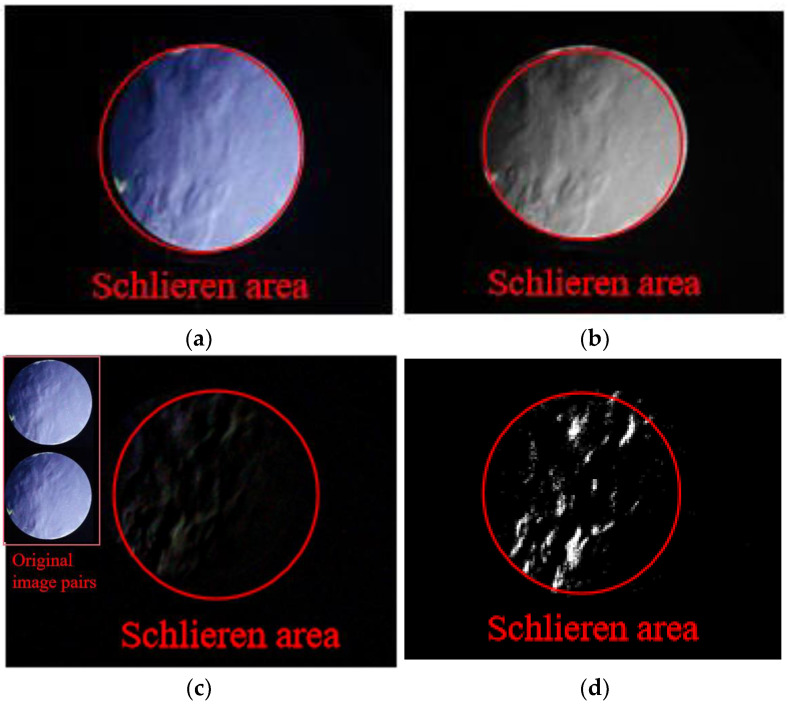
Graphic preprocessing effect: (**a**) a frame of schlieren image (original image); (**b**) image after gray processing; (**c**) motion difference diagram of two frames; (**d**) binarization of motion difference diagram (threshold is 0.1).

The SURF feature matching method has been widely applied in fields such as vehicle speed measurement [26]. Also, the SURF feature matching method is used to measure the speed of cotton flow in combination with Kalman filtering [27]. Many studies have shown that the SURF method has excellent performance in feature point extraction. Multiple groups of locations or areas with the same shape are captured by feature descriptors. The velocity vectors of these location points or areas are calculated by substituting their position information into the corresponding relationship of the fluid image. Different from vehicle motion detection, the relative flow of two gases with density differences is unclear, especially in the laminar flow state from the schlieren image. In addition to removing noise and highlighting fluid flow paths, the motion frame difference image not only preserves the boundary shape of the fluid density gradient but also emphasizes the trajectory of fluid movement. So, the images require pretreatment which is shown in Figure 4 as a sample.

In the preprocessed images in this article, adjacent frame images (approximately 1/30 s), the morphology of the boundary of the fluid density gradient changes slightly. The SURF method will be able to match fluid image microclusters with the same boundary morphological characteristics from motion frame difference images.

This paper applies the pixel movement of the fluid image to directly describe the two-dimensional image of the fluid. Fluid kinematics mainly studies the laws of continuous variation of fluid kinematics, such as velocity, acceleration, etc., with time and space [28]. Based on the description of fluid microclusters by the Eulerian method, the velocity V at point P(x,y,z) in the fluid microcluster is described as $V=V_{x}(x,y,z)i+V_{y}(x,y,z)j+V_{z}(x,y,z)k$. At the same time, the velocity of point Q(x + dx, y + dy, z + dz) expands to point P by the Taylor series:

(3) $$\begin{array}{l} V_{Qx}=V_{x}+\frac{\partial V_{x}}{\partial x}dx+\frac{\partial V_{x}}{\partial y}dy+\frac{\partial V_{x}}{\partial z}dz\\ V_{Qy}=V_{y}+\frac{\partial V_{y}}{\partial x}dx+\frac{\partial V_{y}}{\partial y}dy+\frac{\partial V_{y}}{\partial z}dz\\ V_{Qz}=V_{z}+\frac{\partial V_{z}}{\partial x}dx+\frac{\partial V_{z}}{\partial y}dy+\frac{\partial V_{z}}{\partial z}dz \end{array}$$

According to fluid kinematics, it is described as movement, rotation, and deformation [28]:

(4) $$\begin{array}{ccc} \theta_{x}=\frac{\partial V_{x}}{\partial x} & \varepsilon_{x}=\frac{1}{2}(\frac{\partial V_{z}}{\partial y}+\frac{\partial V_{y}}{\partial z}) & \omega_{x}=\frac{1}{2}\left(\frac{\partial V_{z}}{\partial y}-\frac{\partial V_{y}}{\partial z}\right)\\ \theta_{y}=\frac{\partial V_{y}}{\partial y} & \varepsilon_{y}=\frac{1}{2}(\frac{\partial V_{x}}{\partial z}+\frac{\partial V_{z}}{\partial x}) & \omega_{y}=\frac{1}{2}\left(\frac{\partial V_{x}}{\partial z}-\frac{\partial V_{z}}{\partial x}\right)\\ \theta_{z}=\frac{\partial V_{z}}{\partial z} & \varepsilon_{z}=\frac{1}{2}(\frac{\partial V_{y}}{\partial x}+\frac{\partial V_{x}}{\partial y}) & \omega_{z}=\frac{1}{2}\left(\frac{\partial V_{y}}{\partial x}-\frac{\partial V_{x}}{\partial y}\right) \end{array}\}$$

**Figure 5:**
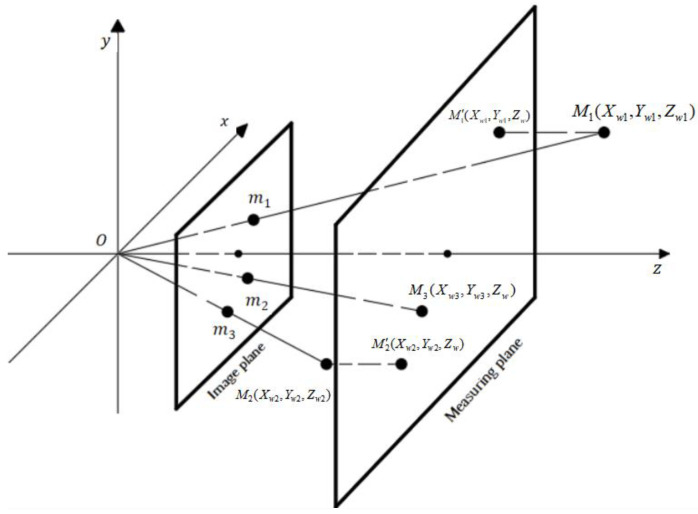
Principle of the pinhole imaging model.

This experiment uses a schlieren system with a single industrial camera to photograph. The quantification of pixel movements uses the classical pinhole model to convert the actual imaging distance [29]. A measurement plane is assumed in the original pinhole model to project the observed fluid image onto the measurement plane. Since the schlieren system observed 2D projection of equal density group movement, the points that are out of the measurement plane are translated to the measurement plane along the *z*-axis direction, as shown in Figure 5. $M_{1}$, $M_{2}$, and $M_{3}$ are the positions of the points in the camera coordinate system; $m_{1}$, $m_{2}$, and $m_{3}$ are the positions of the points on the imaging plane; and $M_{1}{}^{\prime },M_{2}{}^{\prime },M_{3}(M_{3}{}^{\prime })$ are the positions translated to the measurement plane along the *Z* axis.

In this model, $M={\left[X_{w},Y_{w},Z_{w}\right]}^{T}$ are the homogeneous coordinates of actual scene points, and $m={\left[u,\nu \right]}^{T}$ are the homogeneous coordinates on the image frame. The purpose of the calculation is to convert the coordinates of points in an actual scene into pixel coordinates. The point in the world coordinate system is transformed into the camera coordinate system through a rigid body transformation [30].

(5) $$\left[\begin{array}{c} X_{c}\\ Y_{c}\\ Z_{c}\\ 1 \end{array}\right]=\left[\begin{array}{cc} R & t\\ 0 & 1 \end{array}\right]\left[\begin{array}{c} X_{w}\\ Y_{w}\\ Z_{w}\\ 1 \end{array}\right]$$

where ${\left[X_{c},Y_{c},Z_{c}\right]}^{T}$ is the coordinate of the point in the camera coordinate system. R is a rotation matrix, and t is a translation vector. Through basic formula transformation and imaging principle, the 3D coordinate point converts into the pixel coordinate position on the imaging plane. The following relationship can be obtained:

(6) $$\left[\begin{array}{c} u\\ \nu \\ 1 \end{array}\right]=\left[\begin{array}{cccc} f∙f_{x} & 0 & u_{0} & 0\\ 0 & f∙f_{y} & v_{0} & 0\\ 0 & 0 & 1 & 0 \end{array}\right]\left[\begin{array}{c} X_{c}\\ Y_{c}\\ Z_{c}\\ 1 \end{array}\right]$$

where (u,ν) is the coordinate of the pixel coordinate system of the point on the imaging plane. ($u_{0}$,$\nu_{0}$) are the coordinates of the origin of the image coordinate system in the pixel coordinate system. $f_{x}$ is the number of pixels along the *X* axis per millimeter, and $f_{y}$ is the number of pixels along the *Y* axis per mm. f is the focal length of the camera model (unit: mm). ($X_{c},Y_{c},Z_{c}$) are the coordinates of a point in the camera coordinate system. In the actual imaging hardware, the unit pixel lengths in the u direction and ν direction are equal [31]. Therefore, the relationship between the displacement of the scene in the measuring plane and the displacement of the scene in the imaging plane are as follows:

(7) $$\begin{array}{c} \Delta u=F\cdot \Delta X_{c}/Z_{c}\\ \Delta v=F\cdot \Delta Y_{c}/Z_{c} \end{array}\}$$

where F is the pixel number of focal length, Δu is the pixel increment along the u direction, Δν is the pixel increment along the ν direction, $\Delta X_{c}$ is the displacement increment along the X direction in the measurement plane, and $\Delta Y_{c}$ is the displacement increment along the Y direction in the measurement plane. Therefore, the actual velocity of the point in the measurement plane can be expressed by the following equation:

(8) $$V=\frac{\sqrt{\Delta X_{c}{}^{2}+\Delta Y_{c}{}^{2}}}{\Delta t}=\frac{Z_{c}}{F}\cdot \frac{\sqrt{\Delta u^{2}+\Delta \nu^{2}}}{\Delta t}$$

Combined with the fluid kinematics, the point P(x,y,z) pixel coordinates are N(u,v). Then, the virtual two-dimensional expression of the velocity at the point in time t is as follows:

(9) $$\vec{\alpha }=\alpha_{u}(u,v)\vec{i}+\alpha_{v}(u,v)\vec{j}.$$

The velocity of another point $N^{\prime }(u+du,v+dv)$ adjacent to N at the same time is expanded by a Taylor series as follows:

(10) $$\begin{array}{c} \alpha_{Nu}=\alpha_{u}+\frac{\partial \alpha_{u}}{\partial u}du+\frac{\partial \alpha_{u}}{\partial v}dv\\ \alpha_{Nv}=\alpha_{v}+\frac{\partial \alpha_{v}}{\partial u}du+\frac{\partial \alpha_{v}}{\partial v}dv \end{array}\}$$

Due to 3D fluid micro-clusters being captured as 2D images, the motion of the fluid microcluster can only be decomposed into line deformation θ, angular deformation ε, and rotation ω on the virtual plane which is shown in Equation (11):

(11) $$\begin{array}{c} \theta_{u}=\frac{\partial \alpha_{u}}{\partial u},\varepsilon_{u}=0,\omega_{u}=0\\ \theta_{v}=\frac{\partial \alpha_{v}}{\partial v},\varepsilon_{v}=0,\omega_{v}=0\\ \theta_{z}=0,\varepsilon_{z}=\frac{1}{2}\left(\frac{\partial \alpha_{v}}{\partial u}+\frac{\partial \alpha_{u}}{\partial v}\right),\omega_{z}=\frac{1}{2}\left(\frac{\partial \alpha_{v}}{\partial u}-\frac{\partial \alpha_{u}}{\partial v}\right) \end{array}\}$$

**Figure 6:**
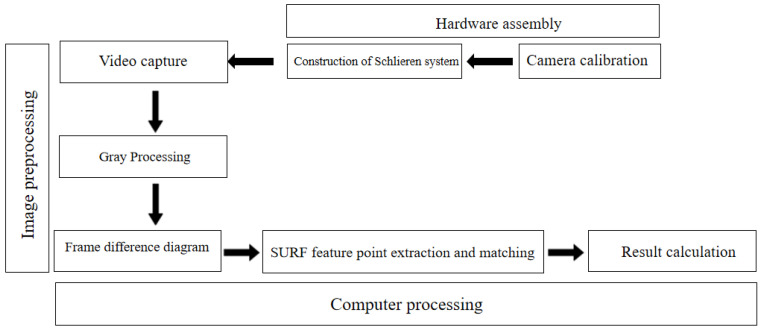
Flowchart of wind speed calculation method.

The two-dimensional pixel representation of fluid motion projection is able to be substituted into the SURF feature matching results to calculate the motion state of adjacent frames of fluid motion. The flowchart of the entire process is shown in Figure 6.

### 2.2. Experiment

#### 2.2.1. Experiment Method

**Figure 7:**
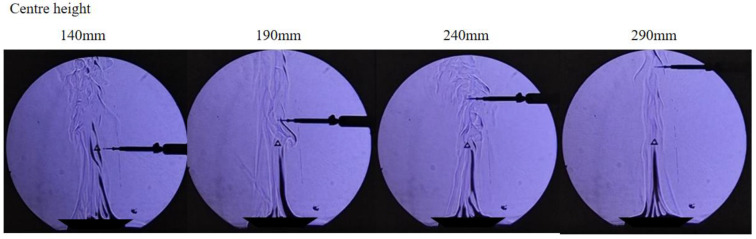
Schematic diagram of sensor arrangement.

The accuracy of this method in measuring velocity distribution and average velocity of thermal turbulent jets is verified through experiments. The experiments consist of two parts. The first part is to determine whether the dimensionless velocity of captured points conforms to the theoretical laws of thermal turbulent jets and the measured values of actual measurement points through the method presented in this article. By adjusting the PWM signal of the fan, the wind speed at the air outlet is kept constant at 1 m/s. In order to exclude the initial segment influence of the jet and the effect of high temperature and humidity on sensors, the wind speed values are measured at heights of 140 mm, 190 mm, 240 mm, and 290 mm from the center of the air outlet and calculate the difference between it and the dimensionless velocity on the central axis. The second part is to verify whether there is a difference between the average wind speed in the area calculated by this method and the sensor measurement value. Meanwhile, the experimental data compare the situation where high-frequency changes in wind speed are captured by the method proposed in this paper. In the experimental process, the average wind speed data from sensors is used as wind speed value. The PWM signal value of fan control changes randomly every 300 milliseconds in 0–255. The wind speed sensors record readings once per second. The PWM value indirectly represents high-frequency changes in wind speed that are unable to be recognized by traditional sensors. The specific layout position is shown in Figure 7.

#### 2.2.2. Experiment Equipment

In order to reduce interference from the external environment, the experiment is conducted indoors. The indoor temperature is 5 °C, and the wind speed is 0–0.1 m/s. The experimental equipment includes a calibrated camera section, a schlieren section, and a thermal turbulent jet generator.

Industrial cameras require pixel measurement after calibration. Zhang’s calibration method is used to calibrate the camera. The software used is MATLAB r2021a. A calibration object with a known structure and high precision is used as the spatial reference. The constraints between camera molding parameters are established through the corresponding relationship between spatial points and hidden image points, and these parameters are solved according to the optimization algorithm [32]. This article selects a 10 × 10 checkerboard calibration template, with each block size of 19 mm × 19 mm. In the experiment, a camera was used to take 86 photos from different angles on the calibration disk, with an image resolution of 1280 × 720. Through the calibration process, the intrinsic matrix of the visual camera is obtained. The calibration intrinsic matrix is as follows:

$$\mathrm{Intrinsic}\;\mathrm{Matrix}=\left[\begin{array}{ccc} f/dx & 0 & u_{0}\\ 0 & f/dy & v_{0}\\ 0 & 0 & 1 \end{array}\right]=\left[\begin{array}{ccc} 1.8326 & 0 & 704.2\\ 0 & 1.8482 & 330.4\\ 0 & 0 & 1 \end{array}\right]$$

**Figure 8:**
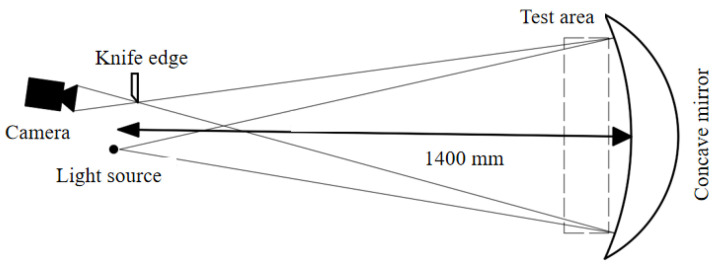
Single mirror off-axis light path schlieren system structure.

A single mirror off-axis light path schlieren system is applied to obtain image information, and the structure is shown in Figure 8. The whole system requires a concave reflector. The light source and camera are arranged on both sides of the central axis of the mirror. The distance between the light source outlet and the center of the mirror is twice the focal length of the concave mirror. The conical light column emitting from the point light source is reflected by the concave mirror, and a spot consistent with the size of the light source is formed on the other side symmetrical to the axis of the mirror, and the light cutting blade is set in this position. Although this simple structure may shade images, it reduces the cost and installation difficulty while meeting the measurement accuracy. All parts are self-assembled after purchase and non-standard parts are 3D printed. The equipment parameters and costs are shown in Table 1.

**Figure 9:**
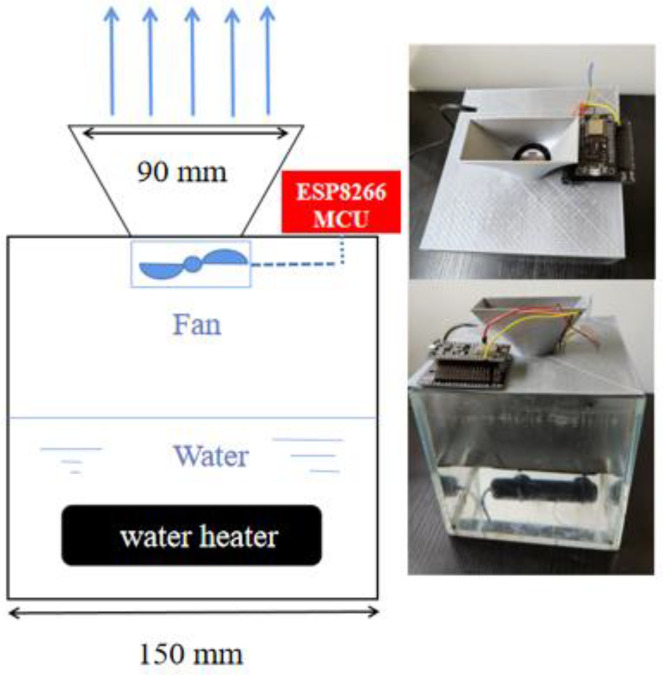
Structure of airflow generator.

The thermal turbulent jet generator includes brushless DC motor fans fixed on the support for 3D printing. Each fan includes 4 pin plug. Since closed-loop control is unnecessary, the pin of the fan is connected to a 5 V power supply, GND, and pulse width modulation (PWM) signal. The PWM pin of each fan is connected to the digital pin of the MCU motherboard for rotation rate control. As shown in Figure 9, water in a 150 mm^3^ water tank and heated to about 60 °C (±5 °C). The air above the water is blown out by the fan accompanied by high temperature and humidity through a 90 mm × 40 mm outlet.

The wind speed measuring instrument uses a SWAMA SWA32 anemometer. In the experiment, the protective cover of the anemometer probe was removed and the probe size is approximately 1.5 mm. The specific performance parameters are shown in Table 2.

## 3. Results and Discussion

### 3.1. Schlieren Image Morphology Analysis

**Figure 10:**
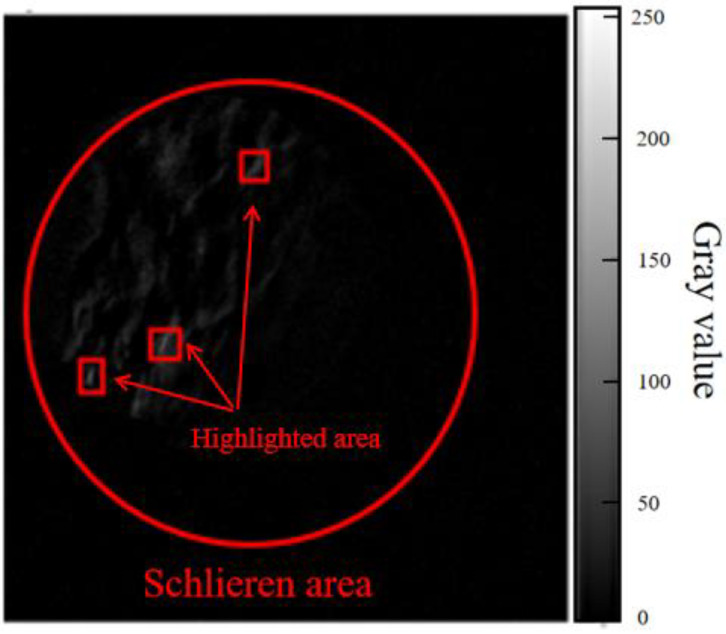
The gray value and its highlighted area of the gray image after frame difference processing.

The obtained image is processed by the software, and the extracted three frames are calculated by frame difference. As shown in Figure 10, although the frame difference image can remove the noncritical information in the image, it also erases part of the image information of fluid flow. The area marked by the red box is the area with the highest gray value of the image. Its gray value is between 220 and 240.

**Figure 11:**
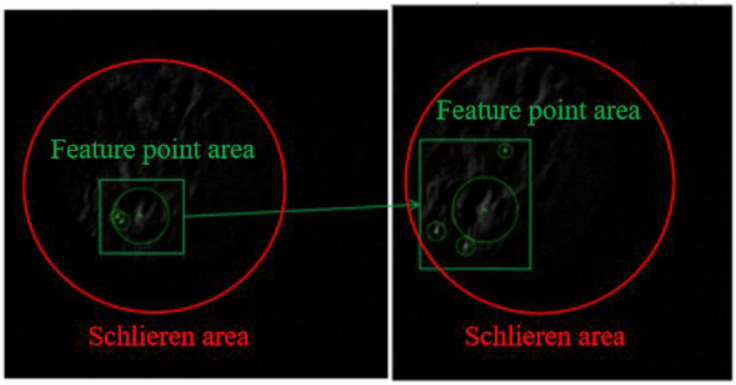
SURF feature points obtained from frame difference images.

If the SURF feature points are extracted directly from the frame difference images, the number of feature points obtained is scarce. Therefore, the correct feature matching points cannot be effectively screened. In Figure 11, two motion-difference images are obtained after the frame difference processing of three adjacent frames. SURF feature points are extracted from two images. Only three feature points are found in the left figure, and only four feature points are found in the right figure. It can be seen from the marked area in the figure that only one area is relatively accurate according to the morphological analysis of the bright part in the figure.

**Figure 12:**
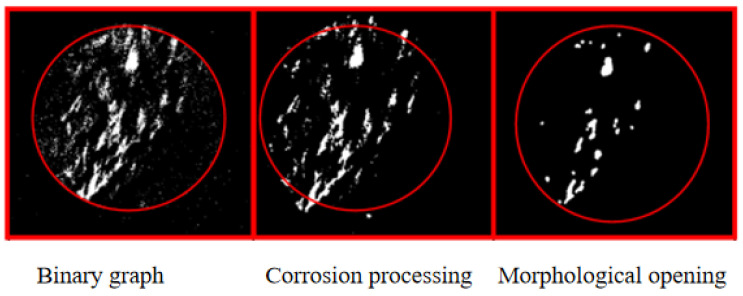
Morphological analysis of the motion difference image.

By binarizing the motion difference images, the outline of the highlighted microclusters is clear on the images. Meanwhile, this method also realizes the two-dimensional projection of the three-dimensional fluid motion state mentioned above. However, although binary images can highlight key information, they also introduce isolated noise to the image. If the image is corroded to achieve the effect of noise reduction, the real details of fluid movement in the image may be covered up, especially the corner or boundary features of the highlighted microclusters, which are shown in Figure 12. Therefore, the images are treated without noise reduction. The following figures illustrate the image changes based on morphology processing. Based on the morphological analysis of motion difference images, binarization can effectively improve image contrast. However, if the image is denoised using an erode or open operation, the boundary of the highlighted clusters will change dramatically. Therefore, it is a relatively reliable method to reduce image noise by controlling the threshold of image binarization.

**Figure 13:**
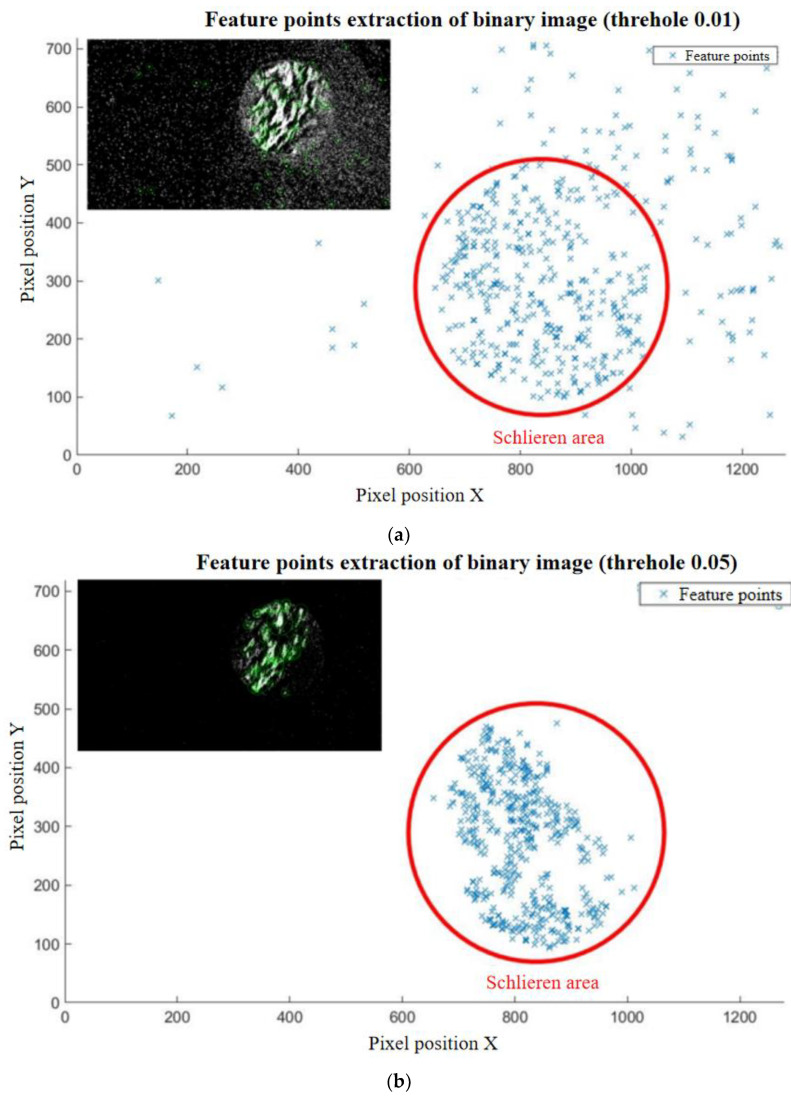

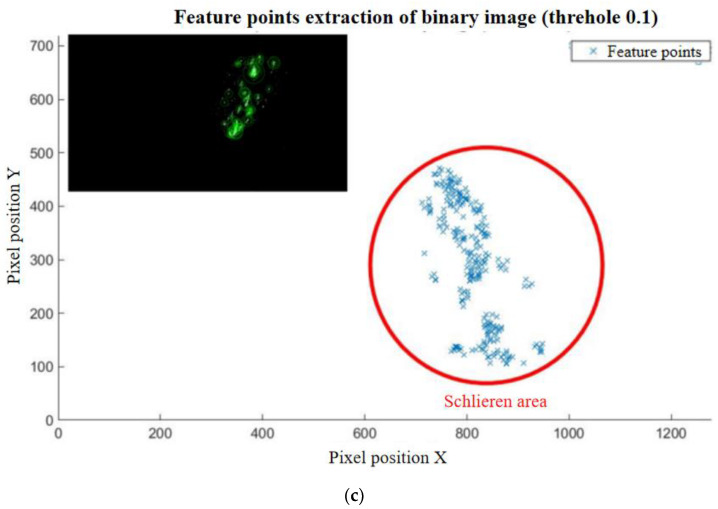
Feature point extraction from binary graphs: (**a**) thresholds = 0.01; (**b**) thresholds = 0.05; (**c**) thresholds = 0.1.

Based on the morphological analysis of motion difference images, binarization effectively improves image contrast. However, if the image is denoised using an erode or open operation, the boundary of the highlighted clusters will change dramatically, and the result is also shown in Figure 13. Therefore, it is a relatively reliable method to reduce image noise by controlling the threshold of image binarization. Choosing an appropriate threshold value for a binary image not only effectively reduces the noise in the image but also reduces the computer’s operating costs compared to image corrosion and open operations. As shown in Figure 13a, when the threshold is 0.01, there are 8228 points extracted by the computer, and the majority are out of the schlieren area. These points outside the schlieren area are not only useless to the test but also introduce errors in later calculations. Therefore, threshold 0.01 is not fit for the test situation. If the threshold is raised to 0.05, as shown in Figure 13b, there are 1077 feature points extracted. Although the noise points still exist outside the schlieren area, the feature points are all included in the schlieren area. Meanwhile, some feature points are out of the flow image in the schlieren area. This result has greatly improved the accuracy of later calculations, but there are still redundant data. To optimize the computational efficiency and decline the number of noise points, the threshold is adjusted to 0.1, as shown in Figure 13c. There are 263 points extracted. This quantity can be contented with later calculation requirements, and all the extracted points are on the flow image. Therefore, a threshold of 0.1 can satisfy not only the purpose of removing noise but also the quantity and quality of feature point extraction.

### 3.2. SURF Feature Point Matching

**Figure 14:**
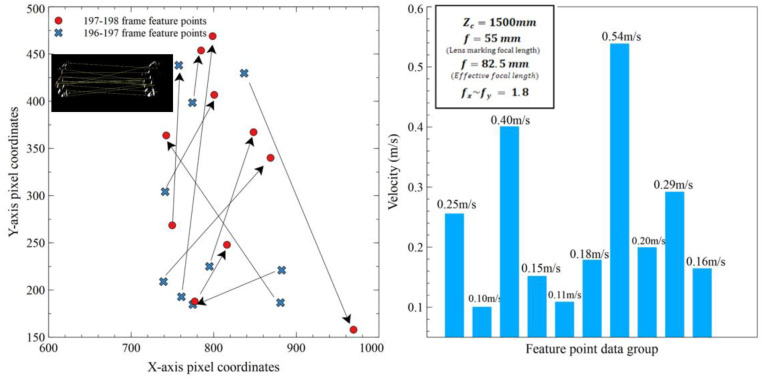
Example of image group feature point calculation.

After selecting the appropriate image processing method by comparison (including motion difference image, binarization, and SURF feature points extraction), the computer performs feature point matching processing on three adjacent frames of images. If the fluid flow images are in a turbulence flow state, the schlieren image of airflow includes a large number of textures. For most images, more than 10 sets of feature matching point groups are obtained in each image group. Moreover, the image sampling rate of this video is 30 fps, and the time difference between the two frames is 0.033 s. Calculated by outputting wind speed values every second, 300 sets of feature matching points will be grasped within one second. After calculating the displacement of these point groups, the average is the displacement of the fluid movement in the image. Taking 196–198 frames of images as an example, ten sets of feature matching points are selected and displacement calculations are performed. The remaining images also follow this process for calculation. The results are shown in Figure 14. The highest velocity is 0.54 m/s, the lowest velocity is 0.1 m/s, and the average velocity is 0.24 m/s.

Since the image sampling rate is 30 frames per second, eliminating individual images with low matching groups can fully satisfy the computational requirements. Table 3 illustrates two examples of one insufficient matching and one redundant matching. As shown in Table 3, one set of feature matching points can be obtained by applying the calculation method used in this paper to frames No. 221 to No. 223 of the collected video. Obviously, this set of data should be excluded because the number of feature matching point groups does not fit the calculation requirements. Meanwhile, the inconformity is associated with the great error in the matching result. Therefore, the elimination of data with matching point groups lower than three groups not only has little impact on the final result but also reduces the error and the adverse impact of wrong matching point groups on the result. Second, image data with more than 10 matching point groups are considered data redundancy such as frames No. 284 to No. 286. This situation should be optimized. To ensure the accuracy of the calculation results, a high frame rate video is required to ensure that the transformation of two adjacent frame images is within a reasonable range. However, the more frames of the image in a video are captured, the more operation pressure is on a computer with the superposition of feature matching point groups. Although 10 groups of data are a tiny quantity for a single step, the amount will be adequate due to the accumulation and superposition of each frame of data. Therefore, the method discussed in this paper adopts two steps to select data. The first step is to remove data from matching points where multiple points correspond to the same point. The second step is to randomly select ten sets of data as the set of feature matching points needed for the calculation. These two simple steps not only delete redundant data but also greatly improve the efficiency of operation automation. In summary, for insufficient matching and redundant matching, the corresponding treatment can basically satisfy the computing requirements.

### 3.3. Wind Speed Estimation Results

#### 3.3.1. Wind Speed Field Measurement

**Figure 15:**
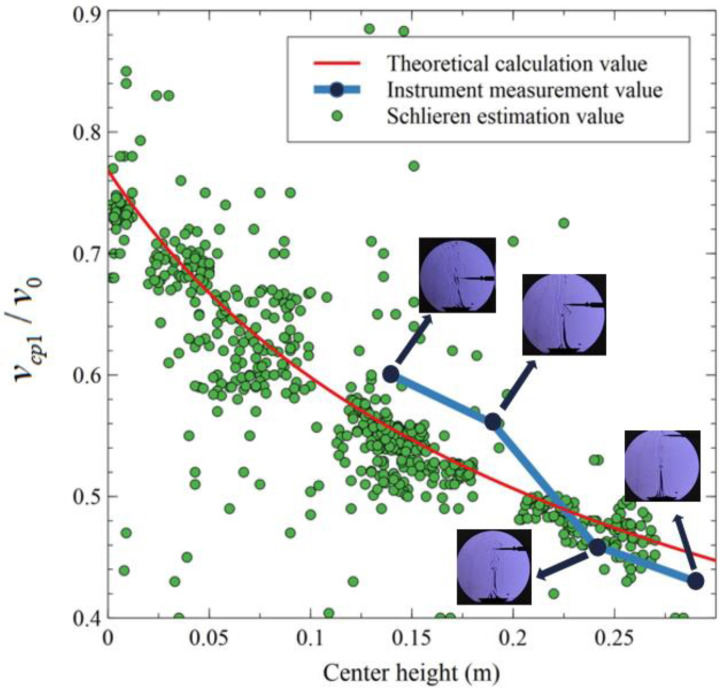
Dimensionless velocity distribution along height direction.

As the experimental model belongs to a turbulent submerged jet, the average cross-section velocity is in accordance with the following equation [33]:

(12) $$\frac{v_{cp1}}{v_{0}}=\frac{0.492}{\sqrt{\frac{aS}{R_{0}}+0.41}}$$

where $v_{cp1}$ is the average velocity in the jet section; $v_{0}$ is the velocity on the air outlet; a is the turbulent coefficient, approximately 0.12; S is the distance of any section from the jet axis to the air outlet; $R_{0}$ is half the width of the air outlet. The experiment records 5 s of the schlieren image. The experimental results are shown in Figure 15. Ideally, a 5 s video will yield 150 frames of images and 1500 groups of matching points. In the experiment, 76 frames of images conform to the computational requirements (including more than 10 matching points) and a total of 760 point groups were obtained, reaching 50.6% of the theoretic number (1500 groups). Among the calculated dimensional velocities, approximately 500 results differ from the theoretical values by within 0.1.

This indicates that most estimation points accurately describe the average velocity at which their cross-section is located. Of course, incorrect matching points still exist, and further research is needed to reduce the number of matching points that may cause errors.

#### 3.3.2. Average Wind Speed Measurement

To verify the accuracy of this method for measuring wind speed, a video was selected as an experimental object. At frames No. 315 to No. 325, the PWM signal value of the fan is 0. This means that the fan is in a short-stop process at this time. In the remaining image frames, the fans operate with random nonzero PWM signals.

This part of the video includes 210 frames with 31 frames of insufficient matching point images and 37 frames of redundant matching point images. Available images account for 85.2% of all images and satisfy the experimental requirement. To compare the calculated value of the feature matching method with the measured value of the sensor, the calculated value of the feature matching method is calculated as an arithmetic average consistent with the time period of the sensor reading.

**Figure 16:**
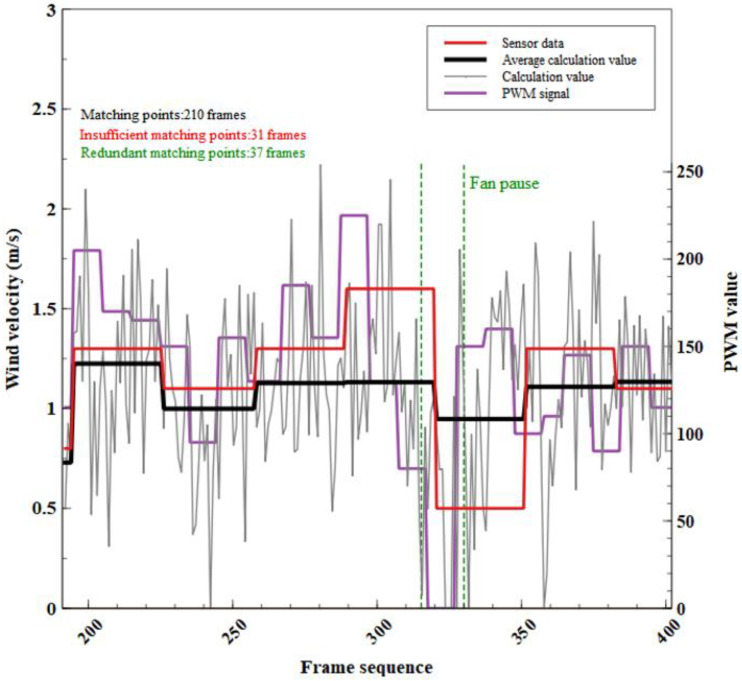
Comparison between sensor data and average calculation value; comparison between the calculated values of each frame and the PWM signal.

As shown in Table 4 and Figure 16, most of the relative errors between the wind speed calculated by the feature matching method and the sensor measurements are less than 15%. The relative error between the calculated frames 194 to 225 and the sensor measurements is the smallest (5.8%). However, because the fan is paused from frame No. 315 to No. 325, there is a large difference between the calculated value of the feature matching method and the measured value of the sensor. Therefore, from frame No. 288 to frame No. 319, the relative error reaches 29%, and from frame No. 319 to frame No. 350, the relative error reaches 90%. However, in the calculation of a single image, a 0 m/s wind speed can be identified. This indicates that the average calculation data does not fully express the wind speed change, especially when the wind speed has a short period of 0 m/s. On the other hand, it shows that the data sampling rate of the feature matching method for calculating wind speed is better than that of this type of sensor. The method also identifies the short-period air flow change with a 0 m/s wind speed rather than filtering the data directly. The rest of the data results are kept within a reasonable relative error range, indicating that the methods discussed in this paper can be applied to wind speed measurement. From the PWM signal change in Figure 14, the trend of wind speed fluctuation calculated from frame-by-frame images is basically consistent with that of PMW signals, which also reflects that the method discussed in this paper is practical and can meet the requirements of measurement.

## 4. Conclusions

This paper investigates how to use SURF feature matching technology in machine vision to perform velocity analysis of turbulent gases with different densities in a schlieren image. This method is helpful for the determination of ventilation speed in winter in agricultural greenhouses. This not only enables quantitative analysis of the speed of the shadow image but also reduces the device cost for practical application. To sum up this appeal, this paper summarizes the following:

1. The three-dimensional flow of two gases with density differences can be projected onto a hypothetical measurement plane and approximated as a two-dimensional flow image by using a shading device and a machine vision calculation method;
2. Combining pixel transformation with fluid kinematics, the relationship between the fluid motion of a two-dimensional shadow image and the change in image pixels is theoretically obtained;
3. The method of removing duplicate image information using a motion difference image and setting a reasonable binarization threshold for fast noise reduction are discussed to achieve more reasonable results of image feature point extraction and to improve the speed of computer operation;
4. The SURF feature matching results of two adjacent images after processing and the calculated wind speed from the results are discussed. The results illustrate that most of the relative errors between the experimental and measured values can be controlled within 15%. When the wind speed is 0 m/s in a short time, the calculation result of this method will be seriously affected. Although the wind speed of 0 m/s can be calculated in the analysis of two adjacent images, it is not reflected in the final output. In future studies, solutions need to be found and improved;
5. In addition to industrial cameras and machine vision software platforms, other parts are completed using open source hardware and 3D printing. The equipment bracket of the shading system is made of a 3D printing device made of PLA material and an open source development board for fan control. The control program can simulate the random wind speed and record the PWM signal through self-programming. The PWM value is also an important reference for the trend of wind speed change;
6. Although the method proposed in this article theoretically estimates wind speed, there are still many factors that cause errors that need to be improved in future research. The complex environment and ever-changing airflow conditions will affect the final calculation results. At the same time, further research is needed to eliminate erroneous calculation points through more effective statistical methods.

## Figures and Tables

**Table 1:** Schlieren equipment parameters and cost.

	Device Parameters	Cost
Concave mirror	Focal length: 700 mm Diameter: 300 mm	USD 60
Knife edge	Tuning range: 0~5 mm Blade length: 30 mm Angle: 0~30° Edge direction: upward	USD 0.5
Light source	Type: LED Diameter: 4 mm Power: 0.5 W	USD 2
Trestle		USD 23
3D printing attachments		USD 10
CCD (include lens)	Interface: USB 2.0 Resolution ratio: 1280 × 720 Frame rate: 30 fps Sensitivity: 0~50 °C 1.8 V/lux-sec at 550 nm Signal-to-noise ratio: 42.3 dB Focal length: 55 mm Effective focal length: 82.5 mm	USD 45
Total cost		USD 140.5

**Table 2:** The features of the sensors.

**SWA 32**	Measurement range: 0.1–10 m/s Measurement accuracy: at 23 °C ± 3 °C ±0.03 m/s at 0.1–0.4 m/s ±0.04 m/s at 0.4–1.33 m/s ±3% read value at 1.33–30 m/s Full operating range 10–45 °C ±0.05 m/s at 0.1–10 m/s Resolution: 0.01 m/s

**Table 3:** Insufficient matching and redundant matching.

Insufficient Matching				Redundant Matching			
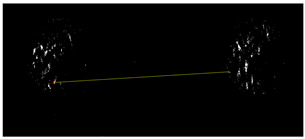				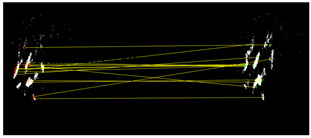			
Matching Point Location				Matching Point Location			
u (221-222)	v (221-222)	u (222-223)	v (222-223)	u (284-285)	v (284-285)	u (285-286)	v (285-286)
815.38263	427.07428	667.79425	371.96356	798.28131	478.50363	799.00092	472.76312
				687.51044	368.84271	770.16553	289.96793
				758.77820	346.49329	731.72919	231.26047
				767.06598	327.19189	695.40533	329.61893
				687.54504	356.99612	695.40533	329.61893
				692.10498	341.34375	693.94812	325.99188
				762.82733	395.07770	785.15594	403.35800
				688.82349	344.70959	740.08984	321.66934
				742.68695	246.68456	837.17480	234.00017
				739.84436	327.85013	759.47424	426.84286
				746.35211	315.70590	690.93073	331.17929
				771.53284	326.28329	690.93073	331.17929
				786.75708	469.63852	854.80908	297.54007
				746.98755	403.53232	795.7077	392.96216
				696.28992	324.26947	695.82886	324.27798

**Table 4:** Comparison of sensor measurement results with calculation results.

Data Sequence	Frame Sequence	Sensor Data (m/s)	Average Calculation Data (m/s)	Relative Error
1	191–194	0.8	0.72	8.8%
2	194–225	1.3	1.22	5.8%
3	225–257	1.1	0.99	9.2%
4	257–288	1.3	1.13	13.3%
5	288–319	1.6	1.13	29.2%
6	319–350	0.5	0.95	90%
7	350–381	1.3	1.10	14.7%
8	381–401	1.1	1.13	3.1%

## Data Availability

The authors confirm that the data supporting the findings of this study are available within the article.
